# The impact of socio-demographic factors on the survival of cancer patients in Zimbabwe

**DOI:** 10.1038/s41598-021-91781-4

**Published:** 2021-06-10

**Authors:** Idika E. Okorie, Ricardo Moyo, Saralees Nadarajah

**Affiliations:** 1 Department of Mathematics, Khalifa University, P.O. Box 127788, Abu Dhabi, UAE; 2 Department of Statistics, University of Zimbabwe, Harare, Zimbabwe; 3 Department of Mathematics, University of Manchester, Manchester, M13 9PL UK

**Keywords:** Cancer, Mathematics and computing

## Abstract

We provide a survival analysis of cancer patients in Zimbabwe. Our results show that young cancer patients have lower but not significant hazard rate compared to old cancer patients. Male cancer patients have lower but not significant hazard rate compared to female cancer patients. Race and marital status are significant risk factors for cancer patients in Zimbabwe.

## Introduction

According to the World Health Organization (WHO), https://www.who.int/news-room/fact-sheets/detail/cancer cancer is a generic term for a wide range of diseases that can affect any part of the body. One unique feature of cancer is the fast development of abnormal cells that over grow their normal boundaries, and which can then attack close or related parts of the body and spread to other organs, the latter process is referred to as metastasizing and it is the major cause of death from cancer.

Cancer is the second leading cause of death globally (WHO), https://www.who.int/news-room/fact-sheets/detail/cancer More than 9 million deaths occurred due to cancer in 2018. The most common cancers are: lung cancer, breast cancer, colorectal cancer, prostate cancer, skin cancer (non-melanoma) and stomach cancer. The most common causes of cancer death are cancers of the: lungs, colorectal, stomach, liver and breast.

There has been little research on cancer in Zimbabwe. Two notable papers are Mandizadza and Rusakaniko^1^ and Kuguyo et al.^2^. Mandizadza and Rusakaniko^1^ emphasize the need to engage an active education campaign to increase public awareness of the significance of cancer in Zimbabwe (although the incidence of cancer in Zimbabwe remains very low compared with the cancer burden in Western countries, see Muguti^3^.) They say that this is particularly important because research show that very late presentation and detection of cancer due to low levels of basic knowledge of cancer symptoms by both patients and health care professionals pose a major challenge in cancer management in Zimbabwe. Mandizadza and Rusakaniko^1^ also point out that little has been done to uncover risk factors, improve diagnosis and treatment of cancer in Zimbabwe. Kuguyo et al.^2^ note that the morbidity and mortality rates of cervical cancer in Zimbabwe are on the increase in spite of the high accessibility of cervical cancer prevention and screening programs in developed countries. This may be due to limited resources as well as the high HIV prevalence in Zimbabwe.

Other papers on cancer in Zimbabwe include: Chokunonga et al.^4^ giving a breakdown of the number of cancer cases recorded among Zimbabweans with respect to year, age, race and gender; Chokunonga et al.^5^ carrying out a survival analysis of 284 cervical cancer patients registered by the Zimbabwe National Cancer Registry; Nkrumah et al.^6^ using a tumour (nephroblastoma) data involving 57 patients over a 3-year period (1984–1987) from Parirenyatwa Hospital in Harare; Katsidzira et al.^7^ comparing the differences in the frequency of colorectal cancer for patients in Zimbabwe according to ethnicity.

We are not aware of any analysis of general cancer data assessing the impact of socio-demographic factors on the survival and hazard rates in Zimbabwe; hence, the motivation of this study. The remaining part of this article contains “Data” in the next section, followed by “Methods' and “Results and discussion” and “Conclusions” sections.

## Data

The data were collected from the Zimbabwe National Cancer registry (ZNCR) which is situated at Parirenyatwa Hospital in Harare. The ZNCR provides specialised cancer management services and data which can be used by health institutions, indigenous and international researchers, lecturers, students, health educators and policy makers for management planning and cancer control programmes. The dates of diagnosis used in the calculation of survival times are from 01 January 2006 to 31 December 2015. The data contain the following variables: age, sex (male and female), marital status (married, divorced, not-known, separated, single and widowed), race (African, African-Albino, Asian, Colour and European), date of diagnosis, status (alive, dead, not-known), date of last contact and survival time (in days). 1452 cancer patients were involved in this study and 909 patients died before the end of the data collection. Actual status of 543 patients was not known at the time of last contact. They were right censored.

The mean age of the cancer patients was calculated as 52.14 and this value was further used as a yardstick to classify the cancer patients as either young (for age < 53) or old (for age $\ge$ 53). Based on this classification, there were 648 old and 804 young cancer patients in this study. The other summary statistics that were calculated includes the minimum age of the patients which was 12, the maximum age of the patients which was 99, the median age of the patients which was 51, the modal age of the patients which was 36, the first quartile of the age distribution which was 41 and the third quartile of the age distribution which was 61. There were 1402 female and 50 male cancer patients. The numbers of patients according to race were African (1413 patients), African albino (1 patient), Asian (5 patients), Coloured (6 patients) and European (27 patients). The numbers of patients according to marital status were divorced (20 patients), married (877 patients), not-known (154 patients), separated (14 patients), single (101 patients) and widowed (286 patients).

**Figure 1:**
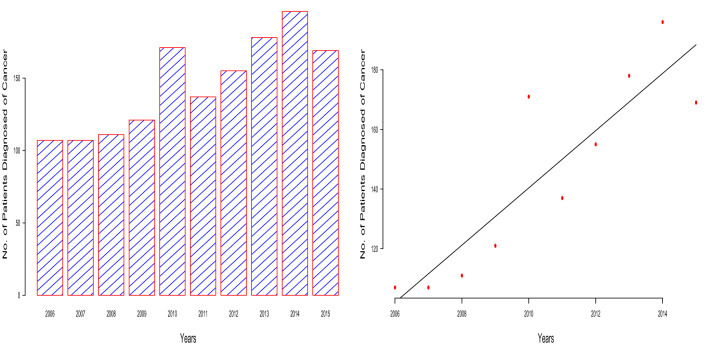
Bar chart of the number of diagnosed cancer patients by year (left). Regression line superimposed on the scatter plot of the number of diagnosed cancer patients versus year (right).

The yearly total of cancer diagnosis is summarized by a bar chart and a scatter plot containing a regression line in Fig. 1. There are 107 cancer patients diagnosed in 2006 and 2007. There are 196 cancer patients diagnosed in 2014, an increase by 58% compared to 2006 and 2007 figures. The fitted regression model is $\widehat{\mathrm{Number}}=-\,19{,}155.6+9.6\, {\mathrm{Year}}$ with $R^2(\text {Adjusted}-R^2)$ of $0.7818\,(0.7545)$ and *p*-value of $6.8\times 10^{-4}$, indicating that cancer incidence is on yearly increase in Zimbabwe.

## Methods

We use the Kaplan–Meier method for survival analysis of the cancer patients data. The Kaplan–Meier is a non-parametric method for estimating the survival probability from the observed survival times^8^. The survival probability at time $t_i$, $S(t_i)$, is computed as

1 $$\begin{aligned} S (t_i) = S (t_{i-1})\left( 1-\frac{d_i}{n_i}\right) , \end{aligned}$$

where $S(t_{i-1})$ denotes the probability of being alive at time $t_{i-1}$, $n_i$ is the number of patients alive just before $t_i$, $d_i$ is the number of patients dead at $t_i$ and $t_0=0$, $S(0)=1$. The *S*(*t*) is a step function that changes value only at the time of each event (death). The Kaplan–Meier survival curve plots *S*(*t*) against time (*t*).

The log-rank test is the most popular non-parametric test for comparing two or more survival curves. The null hypothesis is that there is no difference in survival between the groups. The test compares the observed number of events (deaths) in each group to the expected number of events if the null hypothesis were true. The statistic of the log-rank test is approximately chi-square distributed.

Kaplan–Meier curve only describes the survival according to just one factor under study, but ignores the impact of other factors which may have significant impacts on the survival probability. The Cox proportional hazards regression analysis performs well for both quantitative and categorical variables. It expresses the hazard function denoted by *h*(*t*) at time *t* as

2 $$\begin{aligned} h(t)=h_0(t)\exp \left( \sum _{i=1}^p\beta _i x_i\right) , \end{aligned}$$

where $x_i$’s are *p* covariates, $\beta _i$’s are coefficients measuring the effect size of the covariates for $i=1,2,\ldots ,p$ and $h_0(t)$ is called the baseline hazard corresponding to $x_i=0$ for all *i*. The Cox’s proportional hazards model states that the hazard in one group is $h_0(t)$ and the hazard in the other group is (2). We test the null that $\beta _i$’s are zero.

## Results and discussion

**Figure 2:**
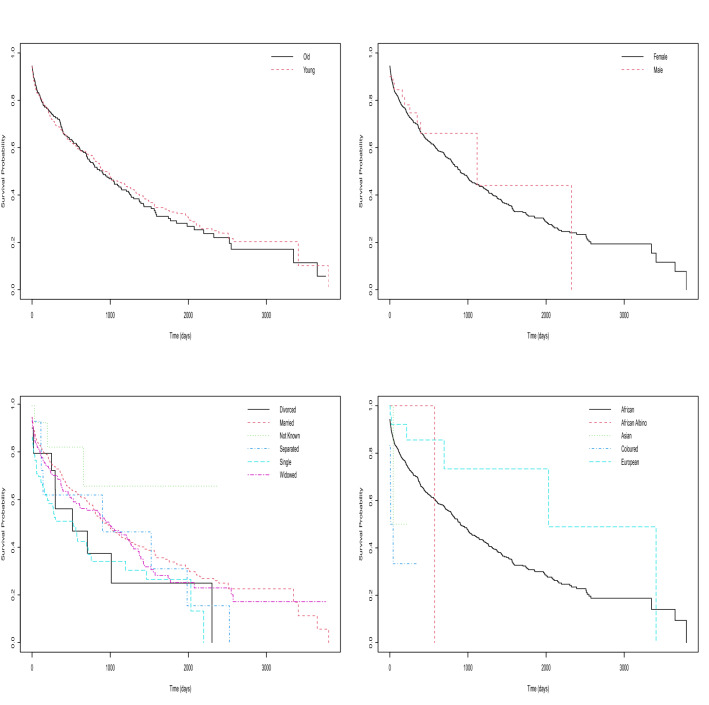
Kaplan–Meier survival plot for the: age of the cancer patients classified as young or old (top left); sex of the cancer patients (top right); marital status of the cancer patients (bottom left); race of the cancer patients (bottom right).

The Kaplan–Meier survival curves for the age of the cancer patients in Fig. 2 show that the survival probability for the young cancer patients mimics that of the old cancer patients. The log-rank test for age gave a *p*-value of 0.80 suggesting that no significant difference exist between the survival curves of young and old cancer patients. However, the median survival time in days for young (old) cancer patients was estimated as 923 (895). There were 2 old cancer patients at risk at 3651 days and there was 1 young cancer patient at risk at 3797 days. The Kaplan–Meier survival curves based on sex of the cancer patients also in Fig. 2 show that the survival probability for the female cancer patients looks similar to that of the male cancer patients and the log-rank test for sex gave a *p*-value of 0.70 suggesting that no significant difference exist between the survival curves of female and male cancer patients. However, the median survival time in days for female (male) cancer patients was estimated as 901 (1117). There was 1 female cancer patient at risk at 3797 days and there was 1 male cancer patient at risk at 2326 days. The Kaplan–Meier survival curves for the marital status of the cancer patients also in Fig. 2 show that the survival probability curves for the divorced, married, not-known, separated, single and widowed cancer patients are mostly widely divergent from one another and the log-rank test for marital status gave a *p*-value of $7.0\times 10^{-5}$ suggesting that the survival curves for the marital status of the cancer patients are generally significantly different from one group to another. However, the median survival time in days based on the marital status of the cancer patients was estimated as divorced (517), married (959), not-known (–), separated (900), single (530) and widowed (985). There was 1 divorced cancer patient at risk at 2304 days, there was 1 married cancer patient at risk at 3797 days, there were 5 not-known cancer patients at risk at 657 days, there was 1 separated cancer patient at risk at 2526 days, there was 1 single cancer patient at risk at 2197 days and there were 7 widowed cancer patients at risk at 2574 days. The Kaplan–Meier survival curves for race of the cancer patients also in Fig. 2 show a wide variation in the survival probability curves for the African, African-Albino, Asian, Coloured and European cancer patients and the log-rank test for race gave a *p*-value of 0.01 suggesting that there are significant differences between the survival curves for different racial groups. However, the median survival time in days for the race of the cancer patients was estimated as African (902), African-Albino (573), Asian (43), Coloured (26) and European (2034). There was 1 African cancer patient at risk at 3797 days, there was 1 African-Albino cancer patient at risk at 573 days, there were 2 Asian cancer patients at risk at 43 days, there were 3 coloured cancer patients at risk at 42 days and there was 1 European cancer patient at risk at 3408 days.

**Table 1:** Parameter estimates of the fitted Cox proportional hazards model (** denotes the *p*-value being less than 1 percent and * denotes the *p*-value being less than 5 percent).

Predictor(s)	${\widehat{\beta }}$	se$( {\widehat{\beta }})$	$\exp ( {\widehat{\beta }})$	$\exp ( -{\widehat{\beta }})$	95% CI	*z*	Pr($>|z|$)
Age::Young	− 0.03599	0.09260	0.9647	1.0366	(0.8045, 1.1566)	− 0.389	0.69752
Sex::Male	− 0.06746	0.27303	0.9348	1.0698	(0.5474, 1.5963)	− 0.247	0.80486
Race::African Albino	0.16200	1.01226	1.1759	0.8504	(0.1617, 8.5505)	0.160	0.87285
Race::Asian	0.42145	1.00297	1.5242	0.6561	(0.2135, 10.8834)	0.420	0.67434
Race::Coloured	1.32161	0.50632	3.7494	0.2667	(1.3899, 10.1145)	2.610	0.00905**
Race::European	− 0.82813	0.41246	0.4369	2.2890	(0.1947, 0.9805)	− 2.008	0.04467*
Marital status::Married	− 0.43307	0.30701	0.6485	1.5420	(0.3553, 1.1837)	− 1.411	0.15837
Marital status::Not-known	− 1.83140	0.58698	0.1602	6.2426	(0.0507, 0.5061)	− 3.120	0.00181**
Marital status::Separated	− 0.16828	0.46890	0.8451	1.1833	(0.3371, 2.1186)	− 0.359	0.71968
Marital status::Single	0.13875	0.33169	1.1488	0.8704	(0.5997, 2.2008)	0.418	0.67572
Marital status::Widowed	− 0.32949	0.31650	0.7193	1.3903	(0.3868, 1.3375)	− 1.041	0.29786

**Figure 3:**
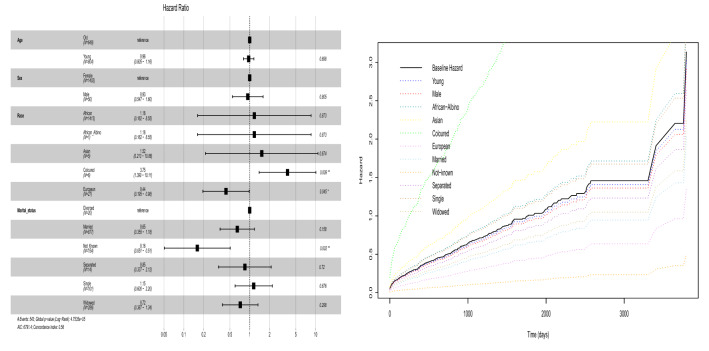
Fitted Cox proportional hazards model indicating the hazard ratio of the cancer patients (left). Comparison of the baseline hazard and hazard of the levels of factors in the Cox proportional hazard model in Table 1 (right).

Table 1 and Fig. 3 make clear that race and marital status are significant. The other variables are not significant. We see that young age decreases the hazard of death due to cancer and the relative risk of death due to cancer for young cancer patients is less (but not significantly) than that for old cancer patients; being male decreases the hazard of death due to cancer and the relative risk of death due to cancer for male cancer patients is less (but not significantly) than that for female cancer patients; being African-Albino, Asian and Coloured increases the hazard of death due to cancer but being European decreases the hazard of death due to cancer; the relative risk of death due to cancer for patients of African-Albino, Asian and Coloured origin is higher (but not significantly except for Coloured and European origins) than that of the patients of African origin; the relative risk of death due to cancer for patients of European origin is quite less than that of patients of African origin; marital status such as being married, not-known, separated, and widowed tends to decrease the hazard of death due to cancer but being single tends to increase the hazard of death due to cancer; the relative risk of death for married, not-known, separated, and widowed cancer patients is generally less (but not significantly except for patients whose marital status are not-known) than that of cancer patients whose marital status are divorced; the relative risk of death for cancer patients whose marital status are single is higher compared to those cancer patients whose marital status are divorced. The likelihood ratio test $p\text {-value}=5.0\times 10^{-5}$, Wald test $p\text {-value}=1.0\times 10^{-4}$ and score (log-rank) test $p\text {-value}=3.0\times 10^{-5}$ indicate that there are significant differences in the survival probability among the levels of each factor in the Cox proportional hazard model.

Furthermore, the differences among the levels of various factors in the model are summarized in Fig. 3. We find that young cancer patients have 3.54% lower hazard than old cancer patients, male cancer patients have 6.52% lower hazard than female cancer patients, African-Albino cancer patients have 17.59% higher hazard than African cancer patients, Asian cancer patients have 52.42% higher hazard than African cancer patients, Coloured cancer patients have 274.95% higher hazard than African cancer patients, European cancer patients have 56.31% lower hazard than African cancer patients, married cancer patients have 35.15% lower hazard than divorced cancer patients, cancer patients whose marital status are not-known have 83.98% lower hazard than divorced cancer patients, separated cancer patients have 15.49% lower hazard than divorced cancer patients, single cancer patients have 14.88% higher hazard than divorced cancer patients and widowed cancer patients have 28.07% lower hazard than divorced cancer patients.

## Conclusions

The hazard ratios (relative risks) in Fig. 3 indicate that young cancer patients have lower but not significant risk of dying compared to old cancer patients. Male cancer patients have lower but not significant risk of dying compared to female cancer patients. African and African-Albino cancer patients are exposed to the same level of not significant risk of dying but Asian cancer patients have higher but not significant risk of dying compared to African and African-Albino cancer patients; Coloured cancer patients have the highest significant risk of dying compared to African and African-Albino cancer patients; European cancer patients have the lowest significant risk of dying than cancer patients from the other racial groups. Cancer patients whose marital status are married, not-known, separated and widowed generally have lower risk of dying compared to divorced cancer patients. However, only cancer patients whose marital status are not-known have significantly lower risk of dying. Single cancer patients are at higher but not significant risk of dying compared to divorced cancer patients. Thus, race and marital status are significant risk factors for cancer patients in Zimbabwe.

Successful cancer treatment largely depends on early intervention. This could explain why Europeans (who are generally wealthier than others and may have access to health facilities in European countries) tend to survive cancer more than others. Europeans may have access for regular screening for different types of cancer to facilitate early detection. Once they are diagnosed they are bound to receive advanced medical treatment. For instance, in countries like Spain, Denmark and the UK, general practitioners (GPs) play the gatekeeping role for access to specialist care and urgent referral pathways are supported by clinical guidelines for patients with suspected symptoms of cancer (see Prades et al.^9^, Probst et al.^10^, Hamilton et al.^11^, NHS England^12^ and NHS England^13^). Such pathways facilitate quick access to specialist opinion and diagnosis within 14 days in the UK (see Hamilton et al.^11^, NHS England^12^ and NHS England^13^) and the first treatment following the GP referral within 62 days (NHS England^12^ and NHS England^13^); hence, leading to shorter time to diagnosis and treatment^14^. Poor Africans on the other hand may not have access to medical facilities which screen for different types of cancer. So, the majority of them receive late cancer diagnosis and only few can afford cancer treatment; thus, leading to little chances of survival. Perhaps, married cancer patients have higher chances of surviving cancer not only because of economic benefits but due to social and emotional support they get from their union; for instance, the marital vow “*...for better, for worse, for richer, for poorer, in sickness and in health,* ...” in Christian weddings. Also, children of such couples may tend to offer social, emotional and even financial support to their parents when they are in perilous situations like battling cancer.

The results in this note could serve as a guide towards sensitization of the risk factors of cancer and help healthcare professionals to improve and design the most effective treatment plan for cancer patients in Zimbabwe.
